# Supplementary material for: Patterns of Intron Gain and Loss in Fungi
Source: PLoS Biol. 2004 Nov 30;2(12):e422. doi: 10.1371/journal.pbio.0020422 (PMC532390; doi:10.1371/journal.pbio.0020422)
Supplement: Table S1 — Also available at http://genes.mit.edu/NielsenEtAl/. (4.3 MB ZIP). [file pbio.0020422.st001.zip › NielsenEtAl/html/531.html]

AN3461.1.NCU05075.1.MG03479.1.FG07875.1


```
 CLUSTAL W (1.82) Multiple Sequence Alignments - Introns Inserted


Sequence 1: NCU05075.1	304 aa
Sequence 2: MG03479.1	301 aa
Sequence 3: FG07875.1	293 aa
Sequence 4: AN3461.1	313 aa
Alignment Length: 322 aa
Number Identitical Residues: 196 aa
Alignment Score (without introns) 8450


MG03479.1 	MAAQPQQQNEKGDAPVAASMK------------K~G~PSALRSIIAGSTAGAVEIV1---
NCU05075.1	MSSAAT----KDDNARAHPAV------------K~G~PSALRSILAGATAGAVEIA1ITY
FG07875.1 	MASTAQ----------PAEKK------------K~Q~PSPLRSVIAGSTAGAIEIV1---
AN3461.1  	MTYQSQIVSEARIQTRSSTSRSSQQTVFNRHISH2G0PSSLRSILAGSTAGAIEIV1---
          	*:  .   ..    . .    ::..:  .   ::   **.***::**:****:**.    

MG03479.1 	----~AKTRTQLNRNLAEGQKLPWPPFGKQWYAGCTTLIIGNSAKAGIR1FVAFDQYKSL
NCU05075.1	PAEF1AKTRTQLNRRLAEGQKLPWPPFGAQWYAGCTTLIIGNSLKAGIR1FVAFDQYKKL
FG07875.1 	----~AKTRSQLNRRLGEGQKLPWPPFGSQWYAGCTTLIIGNAAKAGIR1FVAFDQYKAL
AN3461.1  	----~AKTRSQLNRRLPDSKKLPWPPFGKQWYAGCTTLIIGNSLKAGIR1FVAFDTFKSM
          	     ****:****.* :.:******** *************: ***** ***** :* :

MG03479.1 	LADENGKMSGPRTVLAGFGAGVTESLLAVTPTESIKTTL2--IEDRKSAKPRLRGFLHAV
NCU05075.1	LSDSNGKLSGPGTVLAGFGAGVTESLLAVTPTESIKTTL2--IDDRKSAKPRLRGFLDAV
FG07875.1 	LVDENGKLSGPRTVLAGFGAGVTESLLAVTPTESIKTTL2PSIDDRKSAKPRMRGFLHAV
AN3461.1  	LQDQDGKISGPRTVIAGFGAGFTESLLAVTPFESIKTQL2--IDDRKSANPRMRGFFHGS
          	* *.:**:*** **:******.********* ***** *   *:*****:**:***:.. 

MG03479.1 	PIIARERGIKGFFQGFVPTTARQSANSATRFSAYTFLRQMAESYTAPGEKLGTVGTFAVG
NCU05075.1	PVIYRERGIRGFFQGFVPTTARQAANSATRFSTYTALKQLAESYTAPGEKLGAVGTFAMG
FG07875.1 	PIIARERGLRGFFQGFAPTTARQAANSATRFTAYNFFKQMAESYTAPGEKLGVVGTFAIG
AN3461.1  	GVIFRERGIHGFFQGFVPTTARQAANSATRFSSYTMLKQMAEGYVAPGEKLGTASTFALG
          	 :* ****::******.******:*******::*. ::*:**.*.*******...***:*

MG03479.1 	GVAGLITV~YVTQPLDTIKTR~MQSSEAKSVYGNSFRCASIIFKQEGILTFWSGALPRLA
NCU05075.1	GIAGLITV~YVTQPLDTIKTR~MQSIEAKQLYGNSFRCASIIFKQEGVLTFWSGALPRLA
FG07875.1 	GLAGLITV~YVTQPLDTIKTR~MQSIEARTTYGNSFKCATSIFKQEGVLTFWSGALPRLA
AN3461.1  	GMAGLITV2YVTQPLDTVKTR2MQSLEASKNYKNSFVCAARIFKDEGILTFWSGAVPRLA
          	*:****** ********:*** *** **   * *** **: ***:**:*******:****

MG03479.1 	RLIMSGGIVFTM~YEKSMDLMGRMDPDGKYI
NCU05075.1	RLILSGGIVFTM~YEKSMDLFDRLDPERKYI
FG07875.1 	RLIVSGGLVFTA~YEQIQVWFTKIDPEEKYI
AN3461.1  	RLIMSGGIVFTM2YEKSMDILDSIDPEGRYI
          	***:***:***  **:    :  :**: :**
```
